# Legal Allocation of Design and Construction Risks in Public–Private Partnership Projects: A Comparative Study of the United Arab Emirates, Egypt, and France

**DOI:** 10.12688/f1000research.192125.1

**Published:** 2026-10-20

**Authors:** Alaa Abouahmed, Nout Alahbabi, Alia Alraeesi

**Affiliations:** 1 College of Law, United Arab Emirates University, AL AIN, United Arab Emirates

**Keywords:** Public–private partnerships; construction risk allocation; design liability; construction defects; public contracts; construction disputes; united arab emirates; comparative legal analysis

## Abstract

**Background:**

Public–private partnership (PPP) projects are a principal instrument for delivering infrastructure and public facilities. They combine the private sector’s financing, technical expertise, and operational capabilities with the public entity’s responsibility for ensuring the continuity of public services and the realization of the public interest. The success of these projects depends largely on the precise legal allocation of design and construction risks. Defects may arise from site data, technical requirements, or permits under the control of the public entity. They may also result from detailed design, construction methods, or the performance of subcontractors under the control of the private partner. This distinction assumes particular importance because the effects of a defect may extend from the construction phase to the operation and maintenance phases, leading to delays, increased costs, diminished service levels, and the emergence of claims and disputes.

**Methods:**

The study adopts a comparative analytical approach to design and construction risks in PPP projects. It analyzes legislation, judicial decisions, and scholarly opinions in the United Arab Emirates, Egypt, and France.

**Results:**

The comparison demonstrated a common rule across the United Arab Emirates, Egypt, and France: risk is linked to the party best equipped to foresee and control it, and address its consequences, rather than simply to the party executing the physical works. The findings reveal that the public entity inherently bears the risks of information and decisions under its control. Notably, these include inaccurate site data or existing assets, delayed land handover or utility provision, permits and approvals within its jurisdiction, and modifications imposed on the project’s scope or service levels. Conversely, the private partner bears the risks of detailed design, selection of technology, materials, and execution methods, subcontractor performance, and the achievement of agreed-upon quality, safety, and availability standards.

## 1. Introduction

### 1.1. The practical significance of construction PPP projects

Public–private partnership (PPP) projects are among the most important contemporary mechanisms for delivering infrastructure and public facilities. This is particularly evident in sectors such as roads, transport, energy, water, wastewater, public buildings, and service facilities. Their significance lies in combining the public entity’s responsibility for ensuring service continuity and the public interest with the private sector’s expertise in financing, design, construction, operation, and maintenance. However, these advantages do not arise automatically. The success of a PPP project depends largely on the accurate allocation of risks among the parties, from procurement to contract expiry. ^1^ ^,^ ^2^

Official data from the United Arab Emirates, Egypt, and France confirm that PPP projects have become a practical tool for delivering long-term construction-related projects. In Dubai, the Department of Finance announced a PPP portfolio valued at AED 40 billion, comprising 39 projects for the period 2024–2026. These projects span water, wastewater, waste management, healthcare, roads, parking, public facilities, smart cities, and public transport. In Egypt, the Ministry of Finance announced the approval and commencement of 30 PPP projects valued at EGP 41 billion. A future pipeline of EGP 25–30 billion is also anticipated in sectors such as dry ports, drinking water, wastewater, electricity, and technical education. In France, an official evaluation found that PPP contracts have contributed, since 2004, to the delivery of 248 infrastructure projects, with a total investment of €13.7 billion. Recent policy trends limit their use to projects suited to their long-term contractual nature. ^3^ ^,^ ^4^ ^,^ ^5^

### 1.2. Design and construction risks in long-term PPP contracts

Design and construction risks occupy a central position in construction PPP projects. They represent the point of direct interaction between legal obligations and engineering requirements. Design goes beyond the preparation of drawings and technical calculations; it dictates project constructability, site compatibility, construction costs, safety requirements, quality standards, and operational and maintenance efficiency. Similarly, execution is not limited to physical works; it encompasses material safety, construction methods, specification compliance, time management, quality control, and the delivery of a facility fit for its intended purpose. Consequently, deficiencies in design or execution can lead to delays, cost overruns, change orders, defects, diminished operational efficiency, and the generation of claims and disputes. ^6^ ^,^ ^7^

These risks carry heightened sensitivity in PPP projects compared to traditional construction contracts, given that the contractual relationship in these projects typically spans many years and integrates design, financing, construction, operation, maintenance, and the achievement of specific performance indicators. This is evident in Egypt, where the private sector may undertake design, financing, construction, operation, management, maintenance, and eventual transfer of ownership to the state. It is also evident in France, where a marché de partenariat (partnership contract) may assign the private contractor a comprehensive mission. This may include construction, transformation, or refurbishment, as well as financing, design, maintenance, management, or operation. Accordingly, a design error or construction defect is not confined to the construction phase. It may affect the private partner’s ability to recover its investment, as well as service quality, continuity of the public service, and the economic balance of the contract. ^8^ ^,^ ^9^

### 1.3. Research problem and comparative context

The core problem lies in the fact that certain PPP agreements transfer a broad scope of design and construction risks to the private partner under the assumption that it is the party with superior expertise and capacity for the project’s technical management. However, such a transfer is neither equitable nor effective if the risk stems from data or requirements issued by the public entity, subsequent changes to the project’s scope, deficiencies in preliminary studies, or site conditions beyond the private partner’s ability to foresee or control. Therefore, rational risk allocation must not rely on the absolute transfer of risks; rather, it requires each party to bear the risks it can foresee, prevent, mitigate, price, or insure against at the lowest cost. ^10^ ^,^ ^1^ The comparative analysis of the United Arab Emirates, Egypt, and France reflects diverse PPP and construction contract models. The United Arab Emirates represents a recent model, particularly following Federal Law No. 12 of 2023 on PPPs. Egypt represents an Arab model with practical experience in long-term PPP projects under Law No. 67 of 2010 and its amendments, alongside the continuing role of the provisions of the Civil Law and Administrative Law in governing the liability of project parties. Meanwhile, France offers an advanced model in administrative contracts and PPP contracts, blending the private contractor’s comprehensive mission with the protection of public service continuity and the economic equilibrium of the contract. ^9^ ^,^ ^11^ ^,^ ^12^ ^,^ ^13^

### 1.4. Research gap, aim, and contribution

Despite the importance of risk allocation in PPP projects, much of the literature and contractual practice focuses on financial risks, demand risks, changes in law, force majeure, or early termination. Conversely, design and construction risks—despite their direct nexus to claims, defects, and delays in construction projects—do not consistently receive comparative legal analysis distinguishing between the source of the risk, the phase in which it materializes, and the party bearing its ultimate economic impact. Furthermore, engineering treatment alone does not resolve the decisive legal question: who bears the consequences of a technical error—the public entity, the project company, the designer, the contractor, the consultant, the engineer, or the operator?. ^2^ ^,^ ^14^

This study aims to analyze the legal allocation of design and construction risks in construction PPP projects through a comparative study among the United Arab Emirates, Egypt, and France. It focuses on the procurement and negotiation stages, contract drafting, design, execution, operation, and maintenance. The study seeks to demonstrate how ambiguous risk allocation generates claims and disputes, while proposing practical contractual, legislative, and technical solutions. These include a clear risk matrix alongside precise provisions regarding the public entity’s data, permits, design approvals, change orders, defects, performance tests, and mechanisms for the early resolution of technical disputes. ^2^ ^,^ ^15^

## 2. Methods: Research Design and Jurisdictions

This research adopts a comparative legal analysis methodology concerning design and construction risks in PPP construction projects. This methodology aims to determine the legal basis for allocating each risk, demonstrating its alignment with the risk’s source, the phase of its manifestation, and each party’s capacity to manage it. The analysis is not restricted to identifying the party assigned the risk under the contract; it extends to evaluating the fairness and efficacy of that allocation in light of mandatory legal rules, the nature of the public service, and the requirements for the project’s success throughout its duration. The research adopts a functional criterion whereby risks are borne by the party best equipped to foresee, control, prevent, mitigate, price, or insure against them.

The units of comparison comprise the legal frameworks governing partnerships in the United Arab Emirates, Egypt, and France. The United Arab Emirates was selected as a modern model for regulating partnerships at the federal level, particularly in light of Federal Law No. 12 of 2023 regarding the regulation of partnerships between federal government entities and the private sector. Egypt was selected for its legislative and institutional experience in regulating PPP projects pursuant to Law No. 67 of 2010 and its amendments. France was chosen as an advanced model in administrative and PPP contracts, where public procurement law regulates the marché de partenariat as a comprehensive public contract that may encompass financing, design, construction, operation, and maintenance. ^9^ ^,^ ^11^ ^,^ ^12^

### 2.1. Legal materials and source selection

The research is grounded in the analysis of legislation, regulations, decrees, official manuals governing partnerships and public procurement, relevant judicial precedents and legal principles, government reports, peer-reviewed studies, legal treatises, contractual models, and risk allocation guidelines.

Legal materials and published scholarly references were collected up to July 30, 2026. The research imposed no temporal cutoff on foundational legislation or core judicial precedents, as certain prior rules retain influence in interpreting contracts or illuminating the evolution of the legal system. Nevertheless, priority was given to contemporary sources when analyzing public policies, legislative developments, and practical reports concerning partnership projects. Sources in Arabic, English, and French were utilized to enable direct consultation of original texts and legal doctrine across the jurisdictions under study.

### 2.2. Comparative analytical process

The comparative analysis was conducted across four interrelated stages. In the first stage, the research identified the legal and regulatory rules governing partnership contracts in each jurisdiction, outlining the scope of the party autonomy in risk allocation and the limits of such freedom. In the second stage, design and construction risks were classified according to their source and the phase of manifestation. These risks encompassed deficiencies in preliminary studies, inaccurate site data, ambiguous public entity requirements, design errors or incompleteness, delayed approval of plans, unforeseen site conditions, material and construction method risks, delays, cost overruns, construction defects, failed performance tests, and the lingering impacts of technical errors during the operation and maintenance phase.

In the third stage, the research analyzed how each risk is allocated among the public entity, the project company, the contractor, the designer, the consultant, and the operator, based on applicable legal rules and available contractual documents. The research did not presuppose that the private partner invariably bears all design and construction risks; rather, in each instance, it examined the relevant party’s capacity to foresee the risk, control it, prevent or mitigate its consequences, price it upon tender submission, and insure against it. It likewise examined whether the source of the risk stemmed from information, requirements, or decisions falling within the public entity’s sphere of control.

The fourth stage comprised constructing a comparative risk matrix across the three countries. This matrix correlates the risk type, its genesis phase, the party exercising actual control over it, relevant legal rules, appropriate contractual allocation, and the legal consequence upon its materialization. It also highlights suitable risk management tools, such as site data review, design liability definition, plan approval procedures, change orders, bonds, insurance, performance tests, and recourse to technical expertise or dispute boards.

### 2.3. Scope and limitations

The research accounts for the reality that a risk may originate in one phase while its impact materializes in a subsequent phase. A flaw may stem from insufficient preliminary studies or erroneous site data during the project preparation phase, yet remain concealed until execution, testing, or operation. Consequently, the analysis is not limited to temporal distinctions among phases; it traces the causal nexus between the risk’s source and its contractual, financial, and technical consequences. This approach helps prevent burdening any party with the outcomes of a risk that it could not reasonably foresee or control.

## 3. Results

The comparative analysis shows that the allocation of design and construction risks in PPP projects is not based on a uniform rule assigning all technical risks to the private partner. Instead, the legal systems under study adopt a functional standard. Risk is allocated to the party best able to foresee, control, prevent, mitigate, and price it contractually. Accordingly, the source of the risk—rather than the timing of the defect—is the primary basis for determining ultimate liability and its financial and contractual consequences.

### 3.1. Risks relating to data, permits, and government requirements

The findings show that, as a general rule, the public entity bears the consequences of information, decisions, and requirements within its control. These include site data or existing asset information, land handover, provision of utilities, essential permits or approvals, and changes to the service scope or public facility’s requirements. This risk does not shift to the private partner merely because the contract includes a general obligation of due diligence. This is particularly the case where the relevant information was under the public entity’s control or where the change was introduced after contracting. ^2^ ^,^ ^7^

### 3.2. Controllable design and construction risks

The comparison confirms that the private partner generally bears risks related to detailed design, construction methods, and the selection of materials and technology. It also bears responsibility for managing subcontractors and ensuring quality control, provided that these matters fall within its actual sphere of control. The private partner remains responsible for meeting agreed performance, safety, and quality standards. It must also remedy defects arising from its technical choices or deficiencies in execution. However, this responsibility is not unlimited. It does not extend to risks beyond its control, particularly where they arise from inaccurate public data, subsequent changes in requirements, or unjustified delays in governmental approvals. ^6^ ^,^ ^10^

### 3.3. Public entity’s approval of designs

The findings disclosed that the public entity’s approval of plans does not, as a rule, transfer the responsibility for design safety from the private partner to the entity. Approval typically aims to verify the design’s compatibility with the public facility’s needs and contractual requirements, whereas professional liability for the adequacy of the technical solution and its constructability remains vested in the designer or the project company. Nevertheless, the public entity may bear the consequences of its approval or instructions if it mandates a specific technical solution, implements a substantial modification, or delays approval in a manner that impacts execution duration or costs. ^2^ ^,^ ^16^

### 3.4. Extension of risk to operation and maintenance

The analysis confirmed that design and construction risks in PPP projects do not terminate upon handover or the commencement of operations. A defect may subsequently materialize as diminished facility availability, failed performance tests, increased maintenance costs, failure to meet service indicators, or deductions from the compensation due to the private partner. Consequently, PPP projects necessitate linking design and construction obligations with operation and maintenance obligations, while distinguishing between defects attributable to the private partner’s performance and irregularities originating from requirements or decisions falling within the public entity’s sphere of responsibility. ^7^ ^,^ ^9^

### 3.5. Convergence and divergence among the studied jurisdictions

The United Arab Emirates, Egypt, and France converge in recognizing the importance of prior contractual risk identification and ensuring that the PPP agreement outlines the party obligations alongside the consequences of modifications, delays, and defects. However, France provides a more explicit legal framework governing risk allocation in PPP contracts and its connection to the economic balance of the contract. By contrast, the more recent UAE framework relies to a greater extent on project documentation and the PPP agreement itself. The Egyptian framework combines PPP-specific legislation with general principles of the civil law and administrative contracts. The comparison confirms that effective risk allocation is not achieved by increasing the risks borne by the private partner. It depends instead on the clarity of the contractual risk matrix and its alignment with actual control over each risk. ^9^ ^,^ ^11^ ^,^ ^12^

## 4. Discussion

The research findings reveal that the legal issue concerning design and construction risks does not lie in the existence of the risk itself, but rather in determining the party that bears its consequences upon its materialization. These findings are discussed through the following points.

### 4.1. The significance of distinguishing between the source of the risk and the phase of its manifestation

A defect may materialize during the construction phase, yet originate from an error in preliminary studies, site data, or technical requirements established by the public entity. Conversely, a design error may remain latent until the operational stage or until performance tests fail. Consequently, it is insufficient to identify the phase in which the damage materialized; its technical and legal source must be traced, as the phase of the defect’s manifestation may differ from the phase in which the risk arose or a party breached its obligation. ^1^ ^,^ ^10^

A strand of legal jurisprudence maintains that risk allocation in PPP projects is not founded on transferring the maximum possible degree of risk to the private partner, but rather on assigning the risk to the party best positioned to control the risk’s cause or effectively mitigate its consequences. Where the public entity possesses core information regarding the site, the existing facility, or the functional requirements, burdening the project company with the consequences of the errors contained in such information fails to achieve a sound legal or economic allocation of risks. This principle remains unaffected merely because the contract obligates the company to conduct a reasonable inspection; the public entity retains a standing obligation to disclose the material information it possesses or can obtain during the preparation and procurement stages. ^1^ ^,^ ^2^ ^,^ ^17^

Legal scholarship likewise emphasizes that combining design, construction, and operation within a single project company does not imply that every defect surfacing during the operational phase constitutes a purely operational risk. Reduced performance or failed operational tests may be the direct result of an early design choice or a non-compliant execution method. Therefore, imposing penalties, deducting financial considerations, or burdening the company with remediation costs should not occur prior to determining the root cause of the irregularity both technically and legally, and establishing whether the defect stems from design, execution, facility’s requirements, or an alteration issued by the public entity. ^1^ ^,^ ^18^

The significance of identifying the source of a defect is highlighted in distributing its ultimate cost among the project parties. If a technical report establishes that a defect originated from a design error, the project company or contractor may have recourse against the designer or consultant in accordance with the contract. If it is established that the cause lies in inaccurate site data, delayed permitting, or defective technical requirements issued by the public entity, claims may be brought for a time extension, compensation, or adjustment of the financial consideration. Thus, the source of the defect determines which party ultimately bears its economic impact, rather than merely identifying the party initially demanded by the employer to repair the defect or provide compensation. ^1^ ^,^ ^2^ ^,^ ^10^

The “project company” refers to the independent legal entity established by the investor or private consortium specifically to execute a PPP contract with the public entity and fulfill its obligations. It is frequently designated as a Special Purpose Vehicle (SPV). The SPV may subcontract design, construction, or operation activities to designers, contractors, or operators. However, it remains, in principle, contractually liable to the public entity for project performance. It retains the right of recourse against any subcontractor responsible for a defect under its subcontracts. ^2^ ^,^ ^12^

In the United Arab Emirates, Federal Law No. 12 of 2023 on the regulation of public–private partnerships emphasizes pre-procurement project preparation. This includes feasibility studies, definition of requirements, tender documents, and the project agreement. Accordingly, a defect arising from inaccurate site data or unclear technical requirements is not automatically attributed to the project company. Instead, it requires an assessment of the scope of disclosure by the public entity, the extent to which the company was entitled to rely on such data, and its obligation to raise early warnings regarding any inconsistencies or deficiencies it identifies. ^11^

A recent study on the digital prequalification of contractors supports this trend. It demonstrates that pre-verifying a contractor’s technical, financial, and operational experience mitigates the risk of awarding projects to unqualified entities. However, such qualification does not transfer to the contractor risks arising from data or requirements issued by the administration. Its function is limited to confirming execution capability, while the public entity remains responsible for the accuracy of the information and requirements contained in procurement documents. ^19^

The importance of documentation and disclosure is further enhanced in digitally enabled procurement systems. Electronic traceable records of documents, correspondence, clarification requests, and technical approvals assist in establishing when data were disclosed, when requirements were modified, and when technical inconsistencies were identified. Nevertheless, digital tools do not alter the substantive legal responsibility of the public entity or private partner. The value of electronic records lies in evidentiary clarity, not in correcting inaccurate technical data or reallocating risk automatically. ^20^

The UAE Civil Transactions Law reinforces this distinction. Article 816(3) provides that: “If defects arise or appear during the execution of the work in the materials provided by the employer, or if other factors arise that would hinder the execution of the work in appropriate conditions, the contractor shall immediately notify the employer thereof. If the contractor fails to give such notice, they shall be liable for all consequences resulting from such failure.” Accordingly, the contractor’s liability may arise from failure to notify, even where the underlying cause of the impediment relates to employer-supplied materials or circumstances beyond the contractor’s control. In such cases, liability is based on breach of the duty to warn, rather than on assumption of the original risk. ^21^

The same law further establishes decennial liability. Article 821 imposes liability on both the contractor and the engineer responsible for design or supervision for the total or partial collapse of a structure, or for defects threatening its stability or safety, even where such defects arise from the land itself. Additionally, Article 822 provides that an engineer whose work was limited to preparing the design without being commissioned to supervise the execution shall only be liable for the defects in the designs they prepared. Thus, the source of the defect and the scope of the task assigned to the engineer determine the basis of their internal liability: the design engineer is liable for design defects, whereas the supervising engineer’s liability extends to gross defects associated with the execution works falling within the scope of their supervision. ^21^

In Civil Appeal No. 782 of 2024, the UAE Federal Supreme Court ruled that genuine awareness of a structural defect, its source, and the responsible party is not achieved merely through general indicators or maintenance rumors. Adjudication depends on a definitive technical report determining the defect’s nature, source, severity, remediability, and accountability. This rule affirms that distinguishing between symptom manifestation and technical cause identification is far from theoretical. Instead, it directly affects the commencement of the limitation period for warranty claims and the feasibility of recourse against the contractor or consultant. ^22^

The Federal Supreme Court likewise affirmed, in Commercial Appeal No. 817 of 2024, that a contractor’s warranty obligation regarding execution defects is an obligation to achieve a result rather than an obligation of best efforts. It suffices for the employer to prove that the executed works are defective, unless the contractor proves the damage resulted from force majeure or an extraneous cause beyond its control. In the case underlying the judgment, the dispute involved non-compliant concrete and a resulting reduction in the building’s lifespan. The judgment illustrates that delayed damage manifestation does not exempt the contractor if its nexus to the contractor’s works or utilized materials is established, while maintaining the necessity of technical expertise to determine such nexus. ^23^

In Egypt, decennial liability protects employers from structural collapse and serious defects that threaten stability or safety. Consequently, the employer does not need to prove ordinary fault on the part of the contractor or engineer to invoke this warranty. Article 651 of the Egyptian Civil Code establishes the joint liability of the contractor and the architect for ten (10) years in the event of total or partial collapse, or for defects threatening building stability or safety, even if the cause stems from the land itself. Egyptian civil jurisprudence clarifies that this liability safeguards facility safety as an obligation of result, rather than serving merely as technical accountability for ordinary execution faults. ^24^

Article 652 clarifies the allocation of liability based on the source of the defect. If an engineer prepares the design only, without being commissioned to supervise the execution, they are liable solely for design defects. However, if a defect originates from the execution, liability falls on the contractor, along with the supervising engineer, within the limits of their legal obligations and technical assignment. Therefore, land defects or the manifestation of defects after delivery does not preclude decennial liability for those legally responsible. Nevertheless, determining the defect’s source remains essential to ascertaining who ultimately bears the cost and who has the right of recourse against others. ^24^

Within the framework of Egyptian PPP projects, procurement documents predetermine the technical specifications binding upon the project company. Article 24(c) of Law No. 67 of 2010 mandates that the terms of reference and the tender specifications document include “final product specifications, service specifications and levels, performance indicators, and principal requirements” concerning safety, security, environmental protection, and other standards. Furthermore, Article 34 of the Law mandates that the PPP agreement address responsibility for obtaining licenses, permits, and approvals; the means of quality assurance, monitoring, and supervision; the public administration’s right to modify construction, fitting-out, and operation conditions, together with the bases and mechanisms for compensation; and the principles governing the allocation of certain risks and compensation. ^12^

Article 7 affirms the public administration’s right to modify construction, fitting-out, and development conditions, as well as other works or agreed service fees. These modifications must remain within the limits set out in the PPP contract and require approval from the Supreme Committee for Partnership Affairs. Crucially, this is without prejudice to the project company’s or the administration’s right to compensation in accordance with the principles and rules stipulated by the contract. Furthermore, contracting for PPP projects is subject to the principle of economic and financial equilibrium pursuant to Article 19. Therefore, if the public administration introduces a new technical requirement post-contracting or alters the project’s scope, the matter is treated, principally, as a contractual modification rather than a default by the project company, unless the contract expressly includes such change among the risks accepted and priced by the company upon submitting its tender. ^12^

In France, Article L2213–1 of the Public Procurement Code ^9^ requires PPP contracts to define the terms of risk allocation between the public purchaser and the private contractor. Additionally, Article L2213–3 stipulates that, where the public purchaser entrusts all or part of the design works to the contractor, the implementation terms of the contract must identify the design and supervision team (équipe de maîtrise d’œuvre) responsible for designing the facilities and monitoring their execution. Consequently, French regulation links the definition of design responsibility to explicit risk allocation, leaving no room for subsequent inference upon the emergence of a dispute. ^9^

The official guidelines for PPP contracts emphasize that risk allocation should rest on the nature of the risk, its source, and each party’s capacity to control it. Consequently, imposing a risk on the private partner that it cannot foresee or control typically fails to achieve value for public funds; instead, it prompts the partner to inflate its tender price or demand additional guarantees and compensation. This guideline acquires particular significance in long-term construction projects because the exorbitant pricing of an unmanageable risk can weaken procurement competition and ultimately shift the cost to the public facility or its users. ^16^

This trend is illustrated in the French Council of State’s ruling in Société Sud Terrassement. A public entity contracted for the construction of a solar sludge-drying unit for semi-solid waste generated by a wastewater treatment plant, and the main contractor subsequently subcontracted the excavation works. During execution, soil conditions were found to differ from the pre-contractual geotechnical study, prompting the subcontractor to claim compensation for increased costs. The Council of State ruled that execution difficulties alone do not suffice to warrant compensation—even in a lump-sum contract; rather, the difficulty must be exceptional, unforeseeable, beyond the control of both parties, and disruptive to the general economic equilibrium of the contract. Because the cost increase represented merely 11.3% of the total contract value, the Council of State rejected compensation, affirming that the benchmark is the risk’s impact on the economy of the contract as a whole, rather than solely on the portion assigned to the subcontractor. ^25^

This rule relates directly to the allocation of design and construction risks in PPP projects. Divergent soil conditions, as compared with prior studies, constitute a risk materializing during execution; yet their source may stem from deficiencies in site data or preliminary studies. Nevertheless, the emergence of this risk does not automatically trigger the public entity’s liability for its cost or entitle the project company to compensation. Reference must first be made to the PPP agreement and the risk matrix to ascertain which party assumed this risk, followed by an assessment of its exceptional nature, unforeseeability, and impact on the contract’s economic equilibrium. Therefore, PPP contracts should explicitly stipulate the degree of reliance placed on site data, together with provisions governing time extensions, compensation, or financial adjustment where a material discrepancy arises that the project company could neither foresee nor price. ^2^ ^,^ ^25^

The impact of identifying the source of a defect is demonstrated in recourse among intervening parties under the ruling in Société Bureau Veritas Construction. In a dispute concerning defects emerging after the handover of the regional archives building in Marseille, the Council of State ruled that a technical controller—whose decennial liability is established vis-à-vis the employer—cannot have recourse against the designer, contractor, or others merely upon proof of damage or the absence of personal fault. Rather, the controller must prove each party’s fault and causal contribution to the damage. Furthermore, the controller’s liability toward the employer, as well as its liability in relation to other interveners, is circumscribed by the scope of the mission entrusted to it under the contract. It is therefore clear that the timing of a defect’s appearance is not determinative of liability or recourse. Rather, liability is governed by the origin of the defect, the scope of each party’s assigned role, and the extent to which their conduct contributed to the damage. ^26^

Accordingly, a construction PPP agreement must clearly distinguish among preliminary study and data risks, design risks, execution and material risks, site risks, and performance testing and operation risks. It should likewise stipulate an independent technical mechanism for determining the root cause of a defect, alongside the legal consequences flowing therefrom—such as remediation, time extensions, financial adjustments, compensation, or inter-party recourse. Ambiguity in these matters does not resolve risk; rather, it transforms a technical dispute into a protracted and complex financial claim and legal conflict. ^7^

### 4.2. Legal allocation of risks between the public entity and the project company

The Public Entity’s Liability for Data, Permits, and Requirements Under Its Control.

The public entity’s liability in a PPP project is grounded on the criterion of actual control over the source of the risk. It is inappropriate to saddle the project company with the financial or temporal consequences of data, decisions, or approvals that the company lacks the authority to issue, modify, or independently verify. Assigning these risks to the public entity encourages it to prepare necessary studies and disclose material information prior to procurement, rather than indirectly shifting their cost into bid prices or the project’s financial consideration. ^1^ ^,^ ^10^

Public entity data specifically encompass geotechnical studies and site data, the condition of existing assets and facilities, buried utilities, rights of way, the availability of land and public utilities, and known regulatory or environmental constraints. This does not mean the public entity guarantees every available piece of information; its liability depends on whether the information is material, whether the contract permitted the project company to rely upon it, or whether the entity withheld it despite knowing its impact. Conversely, the project company is bound to conduct a reasonable site inspection and give notice of apparent discrepancies in documents and field conditions. ^2^ ^,^ ^7^

The contractor prequalification procedures support this phase of risk allocation by enabling the public entity to verify tenderers’ technical competence, financial capacity, and operational experience prior to bid submission. However, successful prequalification does not justify transferring risks that are outside the contractor’s or project company’s control, such as inaccurate site data or ambiguous functional requirements. Prequalification proves a tenderer’s capability to execute the works tendered by the public entity; it does not transform the tenderer into a guarantor of information or decisions issued by the entity. ^19^

Regarding permits and approvals, the public entity is responsible for those the project company cannot obtain independently—such as land allocation, essential planning approvals, decisions on making public utilities available, and permits issued by other administrative bodies over which the company has no influence. In such cases, the public entity is obligated to coordinate among competent entities and take the measures upon which the project execution depends. If a permit is delayed or refused due to an administrative decision or a procedural defect falling under the public entity’s control, the event is attributable to it, provided it is established that the delay or refusal caused direct damage to the project and that the project company gave notice thereof within the agreed timeframe. ^2^

This liability likewise extends to functional requirements established for the facility by the public entity. The entity is responsible for defining the required service, performance levels, production capacity, and safety and quality requirements. If post-contracting it transpires that these requirements are deficient or contradictory, or if the public entity issues instructions altering the service scope or adding new works, the matter constitutes a modification of the project obligations rather than a default in execution by the project company. Pursuant to the contract, this warrants a time extension, an adjustment of the financial consideration, or the restoration of the contract’s economic equilibrium. ^16^

Technical data, correspondence, and approval requests acquire additional significance in digital procurement environments. Providing a traceable electronic record of procurement documents, inquiries, clarifications, and change orders helps establish the date of information disclosure, technical requirement modifications, or the notification of the public entity regarding a conflict. Nevertheless, digital documentation does not convert erroneous information into correct information, nor does it automatically transfer risk to the project company; rather, it facilitates identifying the source of the error and the extent of each party’s awareness in the event of a dispute. ^20^

Comparative legal systems endorse this approach. In the United Arab Emirates, Federal Law No. 12/2023 on the Regulation of the Partnership Between the Public and Private Sectors is founded on pre-procurement project preparation. Such preparation includes studies, requirements, tender documents, and the project agreement. In Egypt, Articles 17, 24, and 34 of Law No. 67 of 2010 obligate the administrative authority to review studies, permits, and land allocation prior to procurement. They also require the incorporation of the project information and specifications into the terms of reference, and the regulation of permit responsibilities, compensation for works modifications, and risk allocation within the PPP agreement. In France, Article L2111–1 of the Public Procurement Code ^9^ requires the public purchaser to precisely define the nature and scope of its needs prior to contracting, while Article L2213–1 mandates that PPP contracts define the terms of risk allocation between the purchaser and the private contractor. ^9^ ^,^ ^11^ ^,^ ^12^

Judicial precedent confirms that the mere occurrence of a risk is insufficient to establish the public entity’s liability; rather, its source, impact, and causal nexus must be demonstrated. In *MTD Equity v. Chile*, the arbitral tribunal held that conflicting positions among governmental bodies regarding land use could not transfer the consequences of internal state coordination failures onto the investor. However, it reduced compensation by 50% due to the investor’s failure to exercise due diligence and to align its contractual expectations with the requirement for obtaining necessary planning approvals. In Société Sud Terrassement, the French Council of State rejected compensation claims arising from divergent soil conditions compared to prior studies, as the additional costs did not reach a threshold that disrupted the general economic equilibrium of the contract. Both cases illustrate that public entity liability for data, permits, or requirements does not arise from inaccuracy or delay alone; it requires proof of control, causation, and material impact. ^25^ ^,^ ^27^

To translate these principles into enforceable obligations, the PPP contract should include a dedicated clause specifying: the data whose accuracy is guaranteed by the public entity; information provided for guidance only; the allocation of permits and approvals between the parties and their respective issuance timeframes; the duty to disclose new information; notification mechanisms upon identifying inconsistencies; and the evidentiary standards for financial and temporal impact assessment. This clause should also set out appropriate remedies for breach by the public entity, including time extensions, reasonable cost compensation, adjustments to financial consideration, or orderly termination where the project continuation becomes impossible. Such structuring reduces ambiguity and anchors subsequent negotiations in a clear technical and contractual record rather than post hoc estimation. ^2^ ^,^ ^16^

### 4.3. The private partner’s liability for controllable design and execution

The private partner’s liability is grounded in the criterion of actual technical control over the risk. The project company—whether it executes the works directly or entrusts them to a designer or subcontractor—inherently bears the consequences of decisions falling within the scope of its professional expertise. These include selecting engineering solutions, detailing designs, choosing materials and equipment, scheduling work sequences, managing subcontractors, and overseeing quality and safety. This allocation is justified by the fact that the company is best positioned to prevent such risks, mitigate their consequences, price them, and insure against them when preparing its financial and technical proposal. In addition, verifying the technical competence, financial capacity, and operational experience of tenderers prior to award helps reduce the risk of selecting an entity incapable of executing the project, without extending the company’s liability to risks outside its sphere of control. ^1^ ^,^ ^6^ ^,^ ^14^ ^,^ ^19^

This liability commences at the stage of translating the functional requirements established by the public entity into a constructible and operational design. Where the public entity requires a facility achieving specific production capacity, service levels, and environmental standards, and the project company subsequently selects the structural system, technology, equipment, and drawings necessary to achieve those results, any failure of the chosen system to deliver the agreed performance constitutes, as a rule, a design risk borne by the company. This includes responsibility for interdisciplinary engineering coordination, constructability verification, compliance with applicable laws and technical specifications, and anticipation of operation and maintenance requirements throughout the partnership term. ^2^ ^,^ ^7^

The legal rule remains unchanged where the project company engages an independent designer or subcontractor. The relationship between the company and such entities is internal and does not release the company from its obligations toward the public entity. The company remains the contracting party under the PPP agreement and bears full responsibility for delivering the project in accordance with the agreed specifications and timeframe. The company may subsequently exercise recourse against designers or subcontractors where a defect arises from their fault, provided that its subcontracts incorporate obligations aligned with the PPP agreement—particularly regarding design, quality, testing, warranties, and compensation. ^6^ ^,^ ^28^ An organized electronic log may be utilized to monitor design submissions, the public entity’s comments, and subsequent modifications. Furthermore, entrusting the verification of technical facts to an independent expert or a risk-monitoring system is beneficial in documenting the execution stages and identifying the source of an irregularity at an early stage. ^29^

Execution risks under the private partner’s control include selection of construction methods, procurement of materials, labor and equipment management, coordination among contractors, scheduling, quality and performance testing, and defect remediation prior to handover or during operation. The company likewise bears the consequences of delays arising from poor site management, improper sequencing, subcontractor fault, non-compliant materials, or inadequate testing. It is not sufficient for the company to argue that the works were performed by another party; its obligation toward the public entity is to deliver the facility in accordance with the agreed standard, not merely to exercise best efforts in selecting subcontractors.

Approval of designs, supervision, or inspections by the public entity during the execution does not automatically transfer design and execution risks to it. Such approval generally serves to verify compliance with the project requirements and does not displace the professional liability of the designer or contractor. Liability transfers only where approval includes an express design modification, where the public entity issues binding technical instructions altering the solution chosen by the project company or where it provides a specific guarantee regarding the accuracy of particular designs or data. This distinction prevents the company from invoking routine approvals to evade liability, while also preventing the public entity from using approvals to shift the consequences of its own instructions onto the company. ^7^ ^,^ ^16^

The UAE law reflects this allocation in construction contracts. Article 816(3) of the Civil Transactions Law obligates the contractor to notify the employer immediately if defects appear in materials supplied by the employer or if circumstances arise impeding the execution under proper conditions, and holds the contractor liable for failure to give such notice. Articles 821 and 822 establish a regime of special liability for buildings and structures: the contractor and supervising engineer are liable for collapse or defects threatening structural stability or safety, while the liability of an engineer limited to design is confined to defects in their designs. Accordingly, the private partner must structure its contractual relationships with designers and contractors to ensure defect remediation and effective recourse, without affecting its primary liability toward the public entity. ^21^

In Egypt, Article 2 of the Law on Organizing Participation of the Private Sector in Infrastructure, Services, and Public Utilities projects assigns responsibility to the project company for executing project components, including financing, construction, fitting-out, development, maintenance, and service provision. Article 13 further obligates the company to submit reports on construction, fitting-out, and operational works, as well as compliance with environmental, health, and safety requirements. The Civil Code reinforces this approach. Article 651 establishes liability of the contractor and engineer for collapse or defects threatening structural stability or safety, even where arising from a defect in the land. Article 652 limits the liability of an engineer not tasked with supervision to defects in design only. Accordingly, the project company must clearly define in its subcontracts the scope of each party’s responsibilities and mechanisms of recourse for defects. ^12^ ^,^ ^30^

In France, a PPP contract may entrust the private partner with a comprehensive mission covering design, construction, financing, maintenance, or operation under Article L1112–1 of the Public Procurement Code. ^9^ Where design works are included, the contract must identify the design and supervision team responsible for their execution pursuant to Article L2213–3. Furthermore, entities participating in construction works are subject, within the limits defined by their respective roles, to decennial liability rules for gross defects. This necessitates that the project company not merely designate a designer or technical controller, but explicitly define their actual missions, duties of verification and warning, and the scope of their liability for defects in the contract. ^9^

Judicial application of this principle is illustrated in the French Council of State’s ruling in Société Bureau Veritas Construction. The Council affirmed that a technical controller’s liability is tied strictly to the limits of its accepted mission. If the controller seeks recourse against another party due to a construction defect, it must prove that party’s fault and contribution to the damage, rather than merely proving the absence of its own personal fault. This ruling highlights that in PPP projects, the distribution of works among the project company, designer, contractor, and technical controller does not extinguish any party’s liability. Instead, defining the scope of the mission and technical causality serves as a prerequisite for allocating the ultimate cost of the defect among them. ^26^

The facts of *Malicorp v. Egypt*, concerning a build-operate-transfer (BOT) project, likewise reveal that the core regulatory and financial obligations assumed by the investor constitute independent commitments. These include establishing the project company and providing agreed guarantees, which do not lapse merely due to other difficulties or disputes associated with the project. This confirms that the private partner bears not only physical construction risks, but also the institutional and financing obligations enabling it to execute design, construction, and operation in accordance with the PPP agreement. ^31^

Nevertheless, this liability does not render the private partner an insurer against every risk materializing in the project. The partner does not bear the consequences of the public entity altering the service scope, errors in data provided by the entity, or delays in permits it cannot obtain or influence. This protection applies provided the partner establishes that the event fell outside its sphere of control, that it notified the public entity thereof, and that it took reasonable measures to mitigate its impact. The dividing line is the source of the risk and the company’s realistic capacity to control it, rather than merely the occurrence of damage during the design or execution phase. ^1^ ^,^ ^2^

Consequently, the PPP agreement should explicitly stipulate the project company’s liability for detailed design, execution methods, materials, subcontractors, tests, quality standards, and safety. It should also outline the effect of design approvals, defect remediation mechanisms, and the public entity’s right to reject non-compliant works. Furthermore, the PPP agreement must specify the company’s obligations to remedy such works, required warranties and insurances, and the company’s right of recourse against its subsidiary contractors. Thus, saddling the private partner with risks it can control becomes a means of ensuring the project quality, rather than a mechanism for shifting general or administrative risks beyond its managerial capacity. ^7^ ^,^ ^2^

### 4.4. Effect of design approvals by the public entity

The rule in administrative contracts and in PPP projects related to the operation of public facilities is that the public entity retains the authority to supervise and direct the contract execution within the limits prescribed by law and the agreement. This authority stems from its duty to protect the public facility and public funds and to verify the project company’s compliance with agreed specifications, service levels, quality, and safety standards. Consequently, the public entity may request the submission of plans and designs, review them, express comments thereon, and approve them prior to proceeding with specific execution stages. ^2^ ^,^ ^9^ ^,^ ^12^

A fundamental question consequently arises: Does the public entity’s approval of designs transfer design responsibility to it? As a rule, the answer is in the negative. The public entity’s approval of plans or designs should not be construed as a transfer of design responsibility to it, nor as a release of the private partner from its professional obligations, unless the PPP contract expressly provides otherwise. Administrative approval primarily aims to verify that the proposed design complies with public facility needs, service levels, and the functional requirements established by the public entity. Meanwhile, the soundness of the technical solution, its constructability, and its capacity to achieve the agreed performance remain within the responsibility of the project company and its retained designer. ^2^ ^,^ ^16^

Consequently, a design approval does not preclude requiring the project company to remedy a defect emerging subsequently, if the defect stems from its selection of the structural system, technology, or materials, from an error in calculations and drawings, or from deficiencies in interdisciplinary engineering coordination. Approving a plan does not mean that the public entity redesigned the project instead of the company, nor that it guaranteed the technical results chosen by the designer. Therefore, the contract must explicitly stipulate that the public entity’s review of designs does not diminish the private partner’s responsibility for their accuracy, compliance with laws, standards, and performance requirements, except to the extent that a modification or binding direction is issued by the public entity. ^7^ ^,^ ^2^

However, the effect of approval changes if the public entity exceeds its role of review and supervision by imposing a specific technical solution. If it requests, after design submission, the replacement of a structural system, equipment, or materials selected by the project company, or mandates a material modification in drawings, production capacity, or performance standards, it has exceeded mere design approval. In such cases, it has issued an order to alter the scope of works. The private partner bears responsibility for executing the modification technically. Meanwhile, the contract governs the consequences of the decision regarding cost and time, which may necessitate a time extension, an adjustment of the financial consideration, or the restoration of economic equilibrium, in accordance with the risk matrix and the change clause. ^2^ ^,^ ^32^

Similarly, the public entity’s liability may arise if it refuses to approve a compliant design or delays approval without legitimate cause, provided approval is required to proceed to a subsequent execution stage. In this case, proving the approval delay alone does not suffice. The project company must establish that the design was complete and compliant, that the delay occurred after the expiry of the prescribed or reasonable period, and that it directly caused critical path disruption or genuine cost escalation. The company is likewise bound to give prompt notice, submit an updated schedule, and take reasonable measures to mitigate the impact. Consequently, it is not entitled to compensation for delays that could have been avoided through sound technical management. ^2^ ^,^ ^7^

Digital tools contribute to documenting this relationship, if the contract provides for their use, by maintaining a chronological record of design submissions, requests for clarification, approval or rejection decisions, and change orders. This record minimizes disputes regarding the date of design submission, the scope of modification, or the party that issued it. However, it does not inherently alter the allocation of responsibility for design soundness. The legal value of digital documentation lies in establishing facts and procedures, whereas the determination of liability remains contingent upon the source of the defect, the contract terms, and the scope of direction issued by the public entity. ^20^

In the United Arab Emirates, the public entity’s approval does not alter the professional liability rules prescribed in the Civil Transactions Law. Articles 821 and 822 establish the contractor’s and engineer’s liability for gross defects and restrict the liability of an engineer whose work was limited to design to the defects in their designs. Consequently, the designer or contractor cannot invoke the mere fact of the public entity’s approval of plans to evade liability for a defect that could have been discovered or prevented within the bounds of their professional mission. ^21^

In Egypt, the PPP contract must specify, pursuant to Article 34 of Law No. 67 of 2010, quality assurance means and monitoring and supervision tools. It must also outline the administrative authority’s right to modify construction, fitting-out, maintenance, and operation conditions, along with the bases and mechanisms for compensation. Therefore, design approval constitutes an ordinary supervisory measure so long as it is confined to verifying compliance with procurement documents and the contract. Such approval alone does not transfer design responsibility from the project company to the administrative authority.

However, if the administrative authority prepared the design or imposed a specific technical modification on the project company, and the defect stemmed directly from that design or modification, the company may plead that the defect does not originate from its design or technical selection. In this case, the administrative authority bears the financial and temporal impact of the modification pursuant to the change clause in the contract. Nevertheless, the project company remains bound to give prompt notice if a defect or technical conflict is apparent to it, and to properly execute the approved design or modification. The designer’s liability for design defects under Article 652 of the Civil Code subsists unless it is established that the administrative authority imposed the defective design or modified it in a manner directly causing the defect afflicting the works. ^12^ ^,^ ^30^

In France, if design works are assigned to the private partner in a PPP contract, the contract must identify the responsible design and supervision team. The public entity’s mere review or approval of plans does not alter this; the designer remains responsible for the soundness of their design and the compliance of the works with the contract, technical rules, and legal standards.

The French Council of State has ruled that upon taking delivery of works, the supervising engineer must alert the public entity to defects or irregularities that it could have discovered. If they fail to do so, they may be held liable for breaching the duty of advice. Therefore, the public entity’s approval of the design or acceptance of the project does not release the designer from liability for a fault falling within the scope of their work. ^9^ ^,^ ^33^

Accordingly, the PPP contract should distinguish among four matters: design submission, review, approval, and modification. It must specify the public entity’s review period, the effect of failing to respond, grounds for design rejection, its right to request clarifications, and whether its instructions constitute a change in the scope of works. The contract should also explicitly stipulate that approval does not release the project company from design liability. At the same time, it must affirm the company’s right to time extensions or compensation if the public entity imposes a technical modification or disrupts compliant approvals. Through this drafting, approval transforms into an effective supervisory tool without engendering ambiguity in risk allocation. ^16^ ^,^ ^2^

### 4.5. The interconnection of design and execution risks with the operation and maintenance phase

PPP projects differ from traditional construction contracts because the private partner’s obligation typically does not end upon the completion and handover of the works. In many PPP projects, the private partner is also bound to operate and maintain the facility and provide the service at the agreed standard throughout the contract term. Consequently, a defect originating from design or execution may persist after handover, impairing the facility’s capacity to provide the service and affecting the financial consideration due to the project company. ^1^ ^,^ ^2^

This relationship manifests clearly when an error lies in selecting a specific system, equipment, or materials unsuited to the facility’s actual operation. Following the commencement of operations, this may lead to partial shutdowns of the facility, increased energy consumption, or the need for frequent equipment replacement. Alternatively, the defect may stem from execution, such as improper equipment installation or the use of non-compliant materials. In such cases, substandard performance during operation results from a prior design or execution error, rather than an independent risk arising solely during the operational phase. ^6^ ^,^ ^7^

Availability means that the facility, or the relevant part thereof, is fit for use and capable of providing the service in accordance with the standards specified in the contract. If the facility shuts down or its productive capacity decreases due to a design or construction defect, the public entity may apply a deduction from the periodic consideration or a penalty linked to performance indicators. However, this deduction does not release the project company from the duty to remedy the defect. If the cause of unavailability stems from the company’s design or execution, the company must rectify the cause until the facility returns to the agreed performance level. ^2^ ^,^ ^16^

A distinction must be drawn between financial deductions and defect remediation. The financial deduction compensates the public entity, within the limits specified by the contract, for not receiving the service at the required level. Conversely, defect remediation is an independent obligation resting upon the project company if the defect results from its design or execution. Therefore, the contract must specify whether the public entity has the right to combine the deduction with a demand for remediation, and within what limits. This prevents the imposition of disproportionate penalties while ensuring that deductions do not cease merely because the company has commenced remediation works without actually restoring the facility to the required level. ^2^ ^,^ ^34^

In the United Arab Emirates, the federal PPP framework permits entrusting project stages related to design, construction, operation, and maintenance to the private partner within a long-term contractual relationship. Furthermore, building and structure warranties remain valid, subject to their conditions, even if a defect emerges after operations commence. Therefore, the emergence of a defect during the operational phase does not alter its legal source. If it is established that the irregularity stems from design or execution, the responsible party’s liability subsists, alongside the application of operational and financial consequences stipulated in the PPP contract. ^11^ ^,^ ^21^

In Egypt, Article 2 of Law No. 67 of 2010 makes the project company responsible, depending on the contract scope, for construction, fitting-out, development, maintenance, and service provision. Article 13 likewise obligates the company to submit reports on construction, operation, and maintenance works, as well as compliance with environmental, health, and safety requirements. Combining construction and operation results in saddling the company with the cost of a defect appearing subsequently if it stems from a design or execution default within the company’s control. Consequently, the company cannot plead the completion of the construction phase so long as it remains bound to provide the service and maintain the facility throughout the contract term. ^12^

In France, a PPP contract may entrust the private contractor with a mission encompassing design, construction, maintenance, or operation. The law mandates that the PPP contract define risk allocation and links the consideration due to the contractor to performance objectives specified for each contract stage. Furthermore, maintenance and renewal costs are factored into the project’s economic cost estimation. Thus, French law treats the quality of operation and maintenance as an integral part of the obligations contracted by the public entity, rather than as obligations separate from the quality of design and construction. ^9^

French jurisprudence supports this nexus between the construction phase and the handover and operational phases. In *Office Public de l’Habitat Domanys v. Société SIZ’-IX Architectes*, the Council of State ruled that upon handover, the supervising engineer must alert the public employer to non-conformities that the engineer could have discovered. This duty aims to enable the public entity to refuse the handover, express reservations, and request the remedy of the violation. This rule indicates that in PPP projects, handover tests must verify the facility’s operational readiness and compliance with the standards by which its performance will subsequently be measured. ^33^

Therefore, the PPP agreement should integrate design and construction requirements with operation and maintenance indicators within comprehensive provisions. It is advisable to specify acceptance and operational tests, the availability standard, performance indicators, maximum downtime limits, preventive and corrective maintenance obligations, the deduction calculation mechanism, the defect remediation timeframe, and the distinction between a defect attributable to the project company and an event falling under the public entity’s control. Design, testing, maintenance records, and approval decisions should likewise be archived, as they assist in establishing the source of an irregularity upon the emergence of a dispute. Digital systems can enhance the reliability of these records and facilitate tracking modifications and approvals, without inherently altering the legal liability for the defect. ^2^ ^,^ ^16^ ^,^ ^20^

### 4.6. Comparison matrix for design and execution risk allocation in construction PPP projects

Table 1 illustrates the proposed allocation of design and execution risks in light of the comparison among the UAE, Egypt, and France. This allocation does not represent a rigid rule; rather, it is applied according to the source of the risk, the provisions of the PPP agreement, and each party’s capacity to control it.

**Table 1.** Comparison matrix for design and execution risk allocation.

Risk Type	Party Originally Bearing It	Instances of Risk Transfer or Shared Responsibility	Appropriate Contractual Impact	Comparative Basis
Site data and preliminary studies	The public entity, if the data emanated from it or was within its sphere of knowledge and control.	The project company bears the responsibility for reasonable inspection and for alerting [the entity] to apparent discrepancies.	Extension of the execution time, compensation for additional cost, or adjustment of the financial consideration.	^9^ ^,^ ^11^ ^,^ ^12^
Detailed design	The project company and its retained designer.	Responsibility transfers to the public entity within the limits of the design prepared by it or the technical solution imposed by it.	Remediation of the defect at the company’s expense, or adjustment of cost and time if the defect stems from a binding direction by the public entity.	^9^ ^,^ ^21^ ^,^ ^30^
Design approvals	The project company, because administrative approvals do not release it from design responsibility.	The public entity bears the impact of any technical modification it imposes or any unjustified delay in approving a compliant design.	Retention of the technical responsibility by the company, with its right to compensation or time extension upon modification or delay attributable to the public entity.	^2^ ^,^ ^16^
Permits, site handover, and facilities	The public entity regarding fundamental permits, land allocation, and making public facilities available.	The company bears technical permits associated with the method of execution or the equipment it has chosen.	Time extension or compensation upon delay of a procedure under the public entity’s control.	^2^ ^,^ ^12^
Change orders	The public entity if it alters the service scope, requirements, or productive capacity post-contracting.	The company bears changes inherently falling within the scope of its priced commitments and risks.	Amendment of the contract, time extension, and adjustment of the financial consideration pursuant to the change clause.	^2^ ^,^ ^12^ ^,^ ^32^
Defects affecting operation and maintenance	The project company if the defect stems from design, execution, or maintenance falling under its control.	The public entity bears the impact if the irregularity stems from a modification imposed by it or from data or a decision under its control.	Defect remediation, consideration deduction upon unavailability, or adjustment of commitments depending on the source of the irregularity.	^9^ ^,^ ^11^ ^,^ ^12^

The matrix demonstrates that equitable risk allocation is not based on loading a single party with all consequences. Rather, it relies on distinguishing between risks that the private partner can manage technically and risks that the public entity remains better equipped to manage by virtue of its control over information, permits, or the facility requirements. This matrix can be converted into a practical annex to the PPP agreement, incorporating notice periods, required documents, and the method for estimating cost and time for each risk. ^1^ ^,^ ^2^

## 5. Conclusions

The comparison among the UAE, Egypt, and France demonstrates that the success of construction PPP projects does not depend on transferring the maximum possible level of risk to the private partner. Instead, it depends on allocating each risk to the party best placed to anticipate, control, mitigate, or price it. This approach requires distinguishing between design and execution risks—which, as a general rule, fall within the professional domain of the project company—and risks relating to data, permits, change orders, and requirements falling under the public entity’s control. Accordingly, the source of the risk and each party’s capacity to manage it remain the primary criteria for determining liability and its associated financial and temporal consequences.

The study shows that the project company, as a general rule, bears responsibility for detailed design, the selection of execution methods, materials, and equipment, the management of subcontractors, and compliance with quality, safety, and performance standards. The public entity’s approval of designs does not transfer this responsibility, provided that such approval is limited to verifying conformity with the requirements of the facility and contract. However, if the public entity prepares the design or imposes a specific technical modification, and the defect stems directly from that design or modification, the project company may plead that the defect does not originate from its technical selection. This is without prejudice to the project company’s duty to give notice of apparent defects and to properly execute the directive issued to it. ^2^ ^,^ ^7^

In the UAE, Federal Law No. 12 of 2023 establishes a modern framework for partnerships that can be further developed through the preparation of standardized PPP agreement templates and detailed risk matrices. Such templates should clearly define each party’s responsibilities in relation to site data, design, approval of plans, permits, variations, testing, and operation and maintenance, together with the financial and temporal consequences of each event. This level of detail enhances clarity and contractual certainty in long-term projects. ^2^ ^,^ ^11^

In Egypt, the importance of leveraging accumulated practical experience in long-term PPP projects lies in standardizing procedures for verifying site data, managing change orders, and linking design and construction obligations to operation and maintenance indicators. Law No. 67 of 2010 provides an appropriate foundation by requiring the PPP agreement to define the parties’ obligations, quality control mechanisms, permits, modifications to the works, compensation, and risk allocation. However, the effectiveness of these provisions depends on the precision of procurement and contract documentation for each project, and on clearly assigning responsibility for each technical and administrative element prior to the commencement of execution. ^7^ ^,^ ^12^

The French experience underscores the importance of linking the private partner’s comprehensive role to the economic equilibrium of the contract and the continuity of the public service. The French PPP contract allocates risks between the public purchaser and the private contractor, tying remuneration to performance objectives across the different stages of the contract. It also confirms that transferring risks beyond the private contractor’s control does not yield better value for public funds; rather, it typically results in increased project costs or necessitates the renegotiation of contractual commitments. ^9^ ^,^ ^16^

The study concludes that an effective construction PPP contract must include a clear risk matrix and define, with precision, relied-upon data, allocation of permits, implications of design approvals, variation procedures, acceptance and operational testing, maintenance obligations, and availability and performance indicators. Such precise allocation safeguards public funds and ensures service continuity, while providing the private partner with sufficient clarity regarding the risks it has genuinely assumed and its obligations throughout the project term. ^1^ ^,^ ^2^

## Use of generative AI

Generative AI was used solely for grammar checking, language refinement, and copyediting to improve manuscript readability. All ideas, analyses, and content remain the sole responsibility of the authors.

## Data Availability

No data is associated with this article.
