# Supplementary material for: Legal Allocation of Design and Construction Risks in Public–Private Partnership Projects: A Comparative Study of the United Arab Emirates, Egypt, and France
Source: F1000Res. 2026 Oct 20;15:1737. [Version 1] doi: 10.12688/f1000research.192125.1 (PMC13752442; doi:10.12688/f1000research.192125.1)
Supplement: Peer review history

# Reviewer report: Reviewer response for version 1

**DOI:** 10.5256/f1000research.212078.r533190

**Published:** 2026-11-14

**Authors:** Chethan Ramanna Kumar

**Affiliations:** 1 Amrita Vishwa Vidyapeetham, Mysuru, Karnataka, India

The article looks at who should bear design and construction risks in PPP construction projects in the UAE, Egypt and France. Its central argument is that a risk should sit with the party best able to foresee, control, mitigate, price or insure against it, rather than being passed wholesale to the private partner. The authors argue that what decides liability is where a defect came from, not when it appears. On that basis, the public entity bears risks from its own data, permits and requirements. The private partner bears risks from detailed design, execution methods, materials and subcontractors.

**Strengths**

1. **Relevant and timely topic.** PPP pipelines are growing in all three countries: AED 40 billion in Dubai, EGP 41 billion in Egypt, and the French IGF evaluation. Design and construction risk gets less legal attention than financial, demand or force majeure risk, and the article fills a real gap.
2. **Clear analytical framework.** Separating the source of a risk from the phase in which it shows up (§4.1) is the article’s strongest idea. It explains well why a defect found during operation may really be a design or execution risk. It also explains why availability deductions and the duty to remedy a defect should be treated as separate obligations (§4.5).
3. **Good use of primary legal sources.** The analysis rests on specific legal provisions:
  - UAE: Federal Law No. 12/2023 and the Civil Transactions Law (Arts. 816(3), 821, 822)
  - Egypt: Law No. 67/2010 (Arts. 2, 7, 13, 19, 24, 34) and Civil Code Arts. 651–652
  - France: Public Procurement Code Arts. L1112-1, L2111-1, L2213-1 and L2213-3
4. **Useful case law.** The UAE Federal Supreme Court judgments (Civil Appeal 782/2024, Commercial Appeal 817/2024) and the Conseil d’État rulings ( *Sud Terrassement*, *Bureau Veritas*, *Domanys*) are recent and well chosen. They are explained in a way that shows their practical relevance, for example the point that a definitive technical report is what starts the limitation period.
5. **Balanced position.** The article avoids leaning toward either side. It says plainly that the private partner is not “an insurer against every risk.” It also rejects the idea that a routine design approval moves responsibility onto the public entity.
6. **Strong practical value.** The recommended contract clauses (Section 4.2, and 4.4) and the risk matrix in Table 1 can be used directly by practitioners and contract drafters. The four-step distinction between design submission, review, approval and modification is especially helpful.
7. **Clear structure and writing.** The article follows a logical order through methods, results, discussion and jurisdiction-specific conclusions. The language is good and easy to follow.

**Minor corrections**

1. **Table 1 doesn’t actually compare the three countries.** The “Comparative Basis” column holds only reference numbers. Please show briefly where the UAE, Egypt and France agree or differ for each risk type, for example as a column per country.
2. **Section 3 (Results) relies mainly on international guidance.** It cites World Bank and GI Hub documents (refs 1, 2, 6, 7, 10) more than the national laws and judgments being compared. Please tie each finding to the specific legislation and case law of the three countries.
3. **Some authorities are being applied to PPPs by analogy.** *Société Sud Terrassement* is about an ordinary public works contract and subcontract. *MTD v. Chile* is investment-treaty arbitration. Neither is a PPP dispute, so a sentence noting this limit would strengthen the argument.
4. **Check the UAE Civil Transactions Law citations.** Reference 21 is Federal Decree-Law No. 25 of 2025. Please confirm that Articles 816(3), 821 and 822 match the numbering in the 2025 law and say when it came into force. Please also fix the formatting of that reference: “2025; 816(3): 821–824” reads like volume and page numbers.
5. **Missing Statistical Aspects. **The study is more of theoretically Build, as researcher validation for the theory what we are proposing is important.
6. **Abstract, references and wording:**
  - Add a *Conclusions* section to the abstract, which currently ends at Results.
  - Complete reference 4 (date missing) and reference 2 (“2019th ed”).
  - Put citation numbers in ascending order: “10,1”, “7,2” and “16,2” should be “1,10”, “2,7” and “2,16”.
  - Fix “operator?.” in section 1.4.
  - Replace “subsidiary contractors” with “subcontractors” in section 4.3.

**Recommendation:** Approved, with the minor revisions above.

Is the work clearly and accurately presented and does it cite the current literature?

Yes

If applicable, is the statistical analysis and its interpretation appropriate?

Not applicable

Are all the source data underlying the results available to ensure full reproducibility?

No source data required

Is the study design appropriate and is the work technically sound?

Yes

Are the conclusions drawn adequately supported by the results?

Yes

Are sufficient details of methods and analysis provided to allow replication by others?

No

Reviewer Expertise:

Public-private partnership, sustainable investment, sustainable development.

I confirm that I have read this submission and believe that I have an appropriate level of expertise to confirm that it is of an acceptable scientific standard.

# Reviewer report: Reviewer response for version 1

**DOI:** 10.5256/f1000research.212078.r533184

**Published:** 2026-11-10

**Authors:** Qais Maaitah

**Affiliations:** 1 College of LAW Applied Science University, Bahrain, Bahrain

The manuscript addresses an important legal topic of considerable academic and practical significance, particularly in light of the growing reliance on Public–Private Partnership (PPP) projects and the legal issues arising from the allocation of design and construction risks, together with the associated contractual obligations and liabilities. The comparative approach adopted by the author, examining the legal frameworks of the United Arab Emirates, Egypt, and France, is commendable, as it provides an opportunity to highlight the diversity of legislative approaches and the different legal mechanisms governing such risks. The subject is also particularly relevant to the practical challenges encountered in the implementation of infrastructure projects and the importance of maintaining a fair contractual balance between public authorities and private sector partners. The academic contribution of the manuscript could be further enhanced by developing the comparative analysis, highlighting the distinctive features of each legal system, and incorporating relevant judicial decisions and contractual practices to provide stronger support for the research findings and recommendations.

 Overall, the manuscript represents a valuable contribution to legal scholarship on PPP agreements and construction project risks, addressing a subject that deserves academic attention and further legal examination. Its scholarly contribution could be further strengthened through additional legal analysis and doctrinal elaboration.

  The manuscript is good in terms of the significance of its subject matter and the adoption of a comparative approach, with scope for further development of certain aspects of the legal analysis to enhance its academic contribution.

Is the work clearly and accurately presented and does it cite the current literature?

Yes

If applicable, is the statistical analysis and its interpretation appropriate?

Partly

Are all the source data underlying the results available to ensure full reproducibility?

Yes

Is the study design appropriate and is the work technically sound?

Yes

Are the conclusions drawn adequately supported by the results?

Yes

Are sufficient details of methods and analysis provided to allow replication by others?

Yes

Reviewer Expertise:

Public–Private Partnership Law

I confirm that I have read this submission and believe that I have an appropriate level of expertise to confirm that it is of an acceptable scientific standard.

## References

[1] : Characteristics of Artificial Intelligence in View of Recent Applications.1548: 10.1007/978-3-031-99025-0_44570-576 10.1007/978-3-031-99025-0_44

[2] Addressing Legal and Ethical Considerations of Electronic Trust Certificates in Validating Engineering Projects. *https://ieeexplore.ieee.org/document/10836163* .

[3] Guardians of Privacy: Legal Challenges and Frameworks in the Era of Electronic Personal Data. *https://ieeexplore.ieee.org/abstract/document/10836498* .

# Reviewer report: Reviewer response for version 1

**DOI:** 10.5256/f1000research.212078.r533188

**Published:** 2026-10-30

**Authors:** Jamal Barafi

**Affiliations:** 1 University of Sharjah, Sharjah, United Arab Emirates

The article examines the legal allocation of design and construction risks in PPP projects across the UAE, Egypt, and France, arguing that risk should be borne by the party best equipped to foresee, control, price, and insure against it rather than blanket-transferred to the private partner. It uses a four-stage comparative legal method and concludes that the public entity bears data, permit, and requirement risks, while the private partner bears detailed design, execution, materials, and subcontractor risks, and that design approvals do not transfer design responsibility. The analysis draws on UAE Federal Law No. 12/2023, Egypt's Law No. 67/2010, and Civil Code Arts. 651–652, and French Public Procurement Code provisions and Conseil d'État rulings ( *Sud Terrassement*, *Bureau Veritas*, *Domanys*).

 Although the article already meets the journal’s requirements, a few non-mandatory refinements could further enhance its transparency and readability. In §2.1, a brief clarification of the source-selection process and the principal databases or legislative sources consulted would improve methodological transparency. In §2.2, the rationale for selecting the three countries could be clarified, for example, by highlighting relevant sectoral similarities or the diversity of the legal models examined.

 Finally, in §5, policy recommendations should be explicitly identified using phrases such as “the study recommends” to distinguish them from findings derived from the comparative analysis.

Is the work clearly and accurately presented and does it cite the current literature?

Yes

If applicable, is the statistical analysis and its interpretation appropriate?

Not applicable

Are all the source data underlying the results available to ensure full reproducibility?

No source data required

Is the study design appropriate and is the work technically sound?

Yes

Are the conclusions drawn adequately supported by the results?

Yes

Are sufficient details of methods and analysis provided to allow replication by others?

Yes

Reviewer Expertise:

International Public Law; International Human Rights Law; International Organizations Law; Environmental Law; Public Law.

I confirm that I have read this submission and believe that I have an appropriate level of expertise to confirm that it is of an acceptable scientific standard.
